# Dual-Frequency Piezoelectric Transducers for Contrast Enhanced Ultrasound Imaging

**DOI:** 10.3390/s141120825

**Published:** 2014-11-04

**Authors:** K. Heath Martin, Brooks D. Lindsey, Jianguo Ma, Mike Lee, Sibo Li, F. Stuart Foster, Xiaoning Jiang, Paul A. Dayton

**Affiliations:** 1 Joint Department of Biomedical Engineering, University of North Carolina at Chapel Hill and North Carolina State University at Raleigh, Chapel Hill, NC 27599, USA; E-Mails: khmartin@ncsu.edu (K.H.M.); brooks.lindsey@unc.edu (B.D.L.); 2 Department of Mechanical & Aero-Space Engineering, North Carolina State University, Raleigh, NC 27695, USA; E-Mails: jma9@ncsu.edu (J.M.); sli26@ncsu.edu (S.L.); xjiang5@ncsu.edu (X.J.); 3 Sunnybrook Health Sciences Centre, Toronto, ON M4N 3M5, Canada; E-Mails: hlee@sri.utoronto.ca (M.L.); stuart@sri.utoronto.ca (F.S.F.)

**Keywords:** acoustics, acoustic angiography, dual-frequency, superharmonic, harmonic, broadband, microbubbles

## Abstract

For many years, ultrasound has provided clinicians with an affordable and effective imaging tool for applications ranging from cardiology to obstetrics. Development of microbubble contrast agents over the past several decades has enabled ultrasound to distinguish between blood flow and surrounding tissue. Current clinical practices using microbubble contrast agents rely heavily on user training to evaluate degree of localized perfusion. Advances in separating the signals produced from contrast agents *versus* surrounding tissue backscatter provide unique opportunities for specialized sensors designed to image microbubbles with higher signal to noise and resolution than previously possible. In this review article, we describe the background principles and recent developments of ultrasound transducer technology for receiving signals produced by contrast agents while rejecting signals arising from soft tissue. This approach relies on transmitting at a low-frequency and receiving microbubble harmonic signals at frequencies many times higher than the transmitted frequency. Design and fabrication of dual-frequency transducers and the extension of recent developments in transducer technology for dual-frequency harmonic imaging are discussed.

## 1. Introduction

Fundamentally, ultrasound images are visual representations of the interaction between sound waves and the medium of wave propagation. In ultrasound imaging, an acoustic pulse is transmitted into the field using a transducer capable of producing a temporally short mechanical wave (1–4 cycles) in response to a voltage applied to the transducer. As the incident wave travels into tissue, some of the wave's energy is reflected back toward the transducer by scatterers in the tissue having different acoustic properties (*i.e.*, density and speed of sound) than the background medium. These backscattered acoustic waves are received by the same transducer, which converts mechanical waves into time-varying voltages. These signals are then amplified, digitized, and processed into an image by the ultrasound imaging system. In the most common mode of operation, called “brightness-mode” or “B-mode” ultrasound, grayscale images are formed in which pixel values are proportional to the brightness of scattered acoustic waves. In other system modes, B-mode images are overlaid with colorized maps of blood velocity (color Doppler) or integrated energy from moving scatterers (power Doppler). However, at frequencies typically used in transthoracic and transabdominal ultrasound imaging, blood is a poor ultrasound scatterer which produces echoes approximately a factor of 300 times weaker than surrounding soft tissues [1], making the detection of blood flow in small vessels challenging. For this reason, microbubbles are used as injectable contrast agents, which serve as strong scattering sources and thereby improve imaging of blood flow [2]. While contrast ultrasound is used primarily in cardiology in the United States, it is used more widely in Europe and Asia, and there are substantial ongoing research efforts aimed at evaluating microbubbles as a platform for additional diagnostic and therapeutic applications [3,4]. This review will primarily focus on development of dual-frequency piezoelectric transducers for imaging nonlinear harmonics produced by microbubbles under ultrasound excitation.

### 1.1. Theory of Operation

Microbubble contrast agents are micron-sized shelled gas bubbles that are injected into the vascular space in order to visualize blood flow. When excited by an external acoustic field, microbubbles oscillate non-linearly, producing waves with harmonic content in addition to the fundamental frequency. The degree of harmonic content produced by a single interaction between a microbubble and an acoustic wave increases as the amplitude of the acoustic wave increases, and also increases at frequencies near the resonance frequency of the microbubble [5–8]. Microbubble resonance frequency depends primarily on bubble diameter, though many other physical factors also play a role in determining resonance [9,10]. While microbubbles are strong scatterers that are visible on standard B-mode imaging at the fundamental imaging frequency, the energy arising from microbubbles cannot be separated from that arising from the surrounding tissue. However, by isolating the harmonic signals resulting from microbubble nonlinear behavior it is possible to image microbubbles alone with high specificity.

An effective approach for minimizing tissue echoes in microbubble-specific imaging is to use higher order harmonics. An acoustic wave traveling through tissue generates harmonics due to nonlinear propagation effects, however, most of the energy received at the transducer remains confined to the transmitted frequency (*f_0_*) and the second harmonic (2*f_0_*) [11,12]. Alternatively, microbubble echoes contain higher order harmonics, or “superharmonics,” at frequencies three to 10 times the transmitted frequency [13–16]. In this review paper, we describe transducer technology designed to transmit at low frequencies near microbubble resonance and receive only microbubble harmonics at much higher frequencies, thus spectrally separating microbubble-scattered waves from tissue-scattered waves.

### 1.2. Summary of Commercial Contrast Detection Methods

Currently, most commercial ultrasound systems form images of microbubbles using frequency content within the bandwidth of a single transducer, which is used for both transmitting and receiving. These systems typically reduce tissue echoes by transmitting multiple versions of similar pulses having varying phases and/or amplitudes, then summing received signals from these pulses. Using this process, linear signals originating from tissue cancel while nonlinear microbubble signals sum constructively. In the simplest of these techniques, pulse inversion, a pair of pulses are transmitted which are inverted replicas of one another (*i.e.*, 180° out of phase) [17]. Linear scatterers produce two sets of similarly inverted signals, thus when the received signals from each of the two pulses are added together, the net sum due to linear scattering is zero, assuming no motion has occurred. Because shelled microbubbles vibrate nonlinearly, waves scattered from the contrast agents contain nonlinear components, which do not sum to zero, producing an image of microbubbles alone. Various multi-pulse schemes exist in which the phases and amplitudes of transmitted pulses are altered in order to improve separation of microbubble and tissue signals or to isolate a specific range of harmonics [18–21]. These algorithms are found on commercial ultrasound scanners under names such as Cadence Contrast Pulse Sequencing (CPS) (Siemens) or Power Pulse Inversion (Philips) [22]. The ratio of microbubble to tissue amplitude in an image is known as contrast to tissue ratio (CTR) and is often expressed in dB as a quantitative metric of the effectiveness of a contrast imaging technique. Multi-pulse approaches achieve high CTR at the cost of reduced frame rate and increased susceptibility to motion artifacts. Alternatively, dual-frequency transducers alleviate these problems because their large effective bandwidths allow high CTR imaging using a single pulse.

### 1.3. Design and Fabrication of Piezoelectric Transducers in Diagnostic Ultrasound

#### 1.3.1. Piezoceramic Dimensions

While design theory of piezoelectric transducers is well-covered elsewhere [23,24], basic principles will be reviewed briefly to elucidate challenges relating to dual-frequency transducer fabrication. Transducers used in medical ultrasound consist of a thickness mode resonator. Wave propagation velocity in lead zirconate titanate (PZT) is approximately 4350 m/s [25], resulting in a nominal thickness (λ/2) of 435 μm at 5 MHz, for example, though other factors also affect transducer resonance [26]. In array transducers, grating lobes are avoided by maintaining inter-element separation less than or equal to λ/2. Element width-to-thickness ratio must also be considered, as element aspect ratios determine the acoustic resonance modes [27,28]. Fabrication challenges tend to intensify as feature sizes decrease in all dimensions with increasing frequency. Tradeoffs between desired resonance frequency and element dimensions represent a primary challenge in array design.

#### 1.3.2. Element Boundary Condition Considerations

Matching and backing layers designed with desired acoustic properties are attached in series to the front and back faces of the piezoelectric material, respectively. By reducing the mismatch in acoustic impedance between the piezoelectric material (Z_PZT_ ≈ 34 MRayl) and tissue (Z_water_ = 1.5 MRayl), matching layers increase acoustic transmission into and from the tissue and act as quarter wave transformers. Backing layers improve bandwidth by damping acoustic vibrations at the rear boundary of the piezoelectric material. The loading provided by matching and backing layers also modulates the resonant frequency of the transducer [26]. The primary challenge in design of matching and backing layers is the tradeoff between high sensitivity and broad bandwidth.

#### 1.3.3. Fabrication, Dicing, and Composites

For piezoelectric transducers operating in thickness mode, a “dice-and-fill” approach is commonly used in array fabrication. A wafer-dicing saw cuts and isolates sensing elements, and kerfs resulting from saw cuts are then backfilled to minimize acoustic crosstalk. A dice-and-fill approach is also used to create low-frequency composite materials [29–31] from plates of either piezoelectric ceramics [32] or electrostrictive PMN-PT single crystals [33] by dicing the material and filling the gaps with a polymer [34], then further dicing into individual elements for fabrication of an array. Composite materials can produce better harmonic images than conventional materials because they yield transducers with broader bandwidths (>75%) and improved acoustic matching [35]. Fabrication challenges associated with physical dicing limit acoustic impedance matching in composites.

For harmonic imaging, it may be possible to design a transducer so that both transmission and receiving frequencies are within the bandwidth of a single composite transducer. These composites can be made with 1–3 connectivity, mechanically decoupling the thickness-mode vibration resonance from other undesirable resonances (lateral or elevational modes) [27]. Lateral modes are λ/2 resonances determined by the dimension of the transducer in the lateral direction, or the dimension of image formation. Designing with respect to lateral modes takes on added importance in environments such as intravascular ultrasound (IVUS) in which strict limitations on device size imposed by lumen diameter can create severe aspect ratios (width-to-thickness) that are normally avoided to preserve forward looking directional sensitivity. As an alternative to dice-and-fill methods at higher frequencies, Jiang *et al.*, have demonstrated fabrication of a 40 MHz, 1–3 composite transducer using a plasma etching-based micromachining technique [36]. A 60 MHz IVUS transducer using 1–3 composite has been fabricated and tested on a porcine animal model [35]. These high-frequency broadband transducer materials are promising for imaging microbubble harmonics. The development of high-frequency arrays was greatly facilitated by the introduction of laser micro-machining by Foster *et al.*, a process which has become a standard for commercially-available high-frequency imaging systems [37]. Providing individual electrical interconnects also poses a significant fabrication challenge in high-frequency arrays.

While developments in materials and fabrication have led to diagnostic transducers having increased sensitivity and bandwidth, the use of two independent frequency bands having large separation (at least 3× to 5×) can maximize sensitivity while requiring lower transmit pressures for contrast-specific imaging applications. In the following sections, we describe recent developments in dual-frequency transducer technology.

## 2. Dual-Frequency Transducers

The goal of dual-frequency contrast imaging is to form images of only microbubble harmonics by transmitting acoustic waves at lower frequencies near microbubble resonance (approximately 1–6 MHz) and receiving only higher order harmonic vibrations produced by microbubbles (approximately 10–30 MHz). Researchers have recently demonstrated electrostatic transducers that are capable of encompassing both frequency ranges within a single, extremely broad bandwidth [38,39]. Details of the operation and fabrication of these transducers, commonly referred to as capacitive micromachined ultrasound transducers (CMUTs), are discussed elsewhere [40–42]. CMUTs exhibit inherent nonlinear behavior, which limits their ability to accurately distinguish nonlinear microbubble response, though ongoing investigations attempt to account for these nonlinearities so they may be used for contrast detection reliably [43]. Although CMUTs may eventually demonstrate superior performance to piezoceramics for contrast imaging, they have not yet been demonstrated for this use *in vivo.* While researchers continue to investigate ultra-wideband electrostatic transducers as well as approaches for increasing the bandwidth of piezoelectric transducers [35,36,44,45], this review will primarily focus on devices that use separate transducers for transmission and reception and thus allow for independent design of the two transducers to achieve desired characteristics.

### 2.1. Imaging Microbubble Contrast Agents

**Figure 1.**
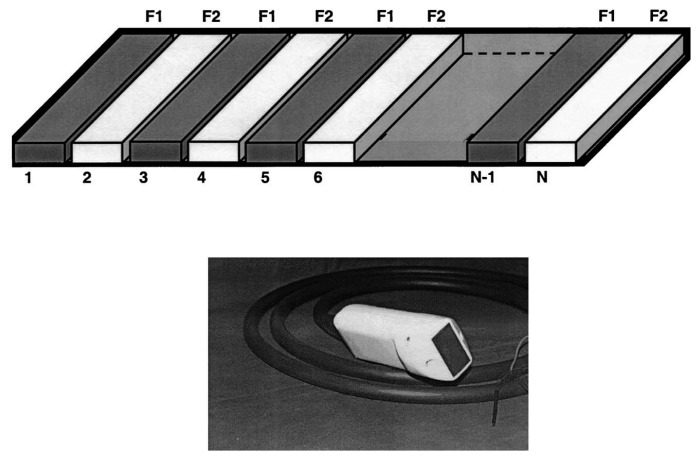
Schematic of the first design incorporating dual-frequency transducers for the purpose of contrast detection (**top**). Odd numbered elements had a center frequency of 2.8 MHz with a fractional bandwidth of 80% while the even elements had a center frequency of 0.9 MHz with a fractional bandwidth of 50%. Odd numbered elements were used for imaging superharmonics generated by nonlinear vibrations of microbubbles excited with a low-frequency pulse provided by the even elements. A photograph of the actual transducer is shown after the elevational lens has been added (**bottom**). Figure reprinted with permission from [46].

Bouakaz *et al.* first reported imaging of third and fourth harmonics of microbubbles in 2002 in experiments which used a 96-element array with interleaved 0.9 MHz transmission elements (50% bandwidth) and 2.8 MHz receiving elements (80% bandwidth) [46–48]. Using this probe with a commercial imaging system, the authors demonstrated the ability to image microbubbles while rejecting tissue signals *in vivo* [47] (Figure 1). While interleaving low and high-frequency elements yields dual-frequency transducer arrays with smaller form factors relative to arrays stacked in the elevation direction, grating lobes were introduced due to an increase in inter-element separation, and signal-to-noise ratio (SNR) was diminished due to reduction in receiving area and in the number of received signals contributing to the beamformed signal.

In 2005, Kruse and Ferrara demonstrated the use of two piston transducers with a wide bandwidth separation for imaging microbubbles using a transmission frequency of 2.25 MHz (70% bandwidth) and a receiving frequency of 15 MHz (66% bandwidth) [49]. Wide separation between the two frequencies ensured high CTR due to the low amplitude of higher order tissue harmonics, while a high receiving frequency produced high-resolution images. In more recent studies, Ferrara's group has designed transmit low/receive high (TLRH) arrays with two outer rows of 64 elements transmitting at 1.48 MHz, and a central row of 128 elements receiving at 5.24 MHz [50]. In addition to harmonic imaging of microbubbles, the high-frequency row of this three-row array was used to deliver a long (100 cycle), low-amplitude (200 kPa peak negative pressure) “pushing” pulse for radiation-force enhanced adhesion of targeted microbubbles for molecular imaging [51,52]. This work has recently been extended to 3D molecular imaging [53]. Subsequent generations of this array featured low-frequency rows capable of delivering either broadband, high peak pressure waveforms for cavitation-mediated therapy or narrower band, high time-average power waveforms for thermal therapy [54,55]. A similar three-row array with a central row of 128 elements operating at 1 MHz (90% bandwidth) and elevationally-aligned outer rows of 128 elements operating at 10 MHz (90% bandwidth) was constructed by Vermon (Tours, France) [56].

In 2010, van Neer *et al.* compared designs for interleaved and multi-row arrays [57,58]. Designs with interleaved elements having high ratios of receive to transmit elements (*i.e.*, at least three receive elements per transmit element) were capable of producing beams with reduced distortion artifacts and tighter −6 dB beam widths relative to two- or three-row arrays. By greatly increasing the number of receiving elements, grating lobes of interleaved designs were limited to −40 dB and high SNR was ensured. However, it should be noted that arrays with interleaved elements of different frequencies cannot be manufactured using standard dice-and-fill approaches from a single piezoelectric plate without significant alteration to manufacturing processes (see “Design and Fabrication” section).

In spite of the promise shown by several of these dual-frequency arrays, transition towards transducers with higher receiving frequencies has been accompanied by several fabrication challenges. Because standard array production techniques faces difficulties scaling to higher frequencies [59], high-frequency transducers can utilize mechanical steering of a single focused element in lieu of an array. Many of these focused single-element transducers have been possible due to the use of flexible piezoelectric composites rather than inflexible piezoelectric ceramics [60,61]. An important advancement for high-frequency arrays has been the development of composites with large triangular pillars to suppress lateral modes while maintaining high sensitivity [62,63].

**Figure 2.**
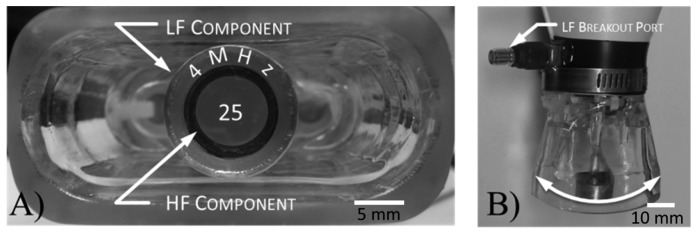
The mechanically steered dual-frequency transducer is composed of a central high-frequency (25 MHz) spherically focused piston transducer inserted into an annular, confocally aligned low-frequency transducer (4 MHz). (**A**, End-on view) Harmonic imaging is performed by mechanically sweeping the arm while transmitting on the outer element and receiving on the inner element. (**B**, Side view)

**Figure 3.**
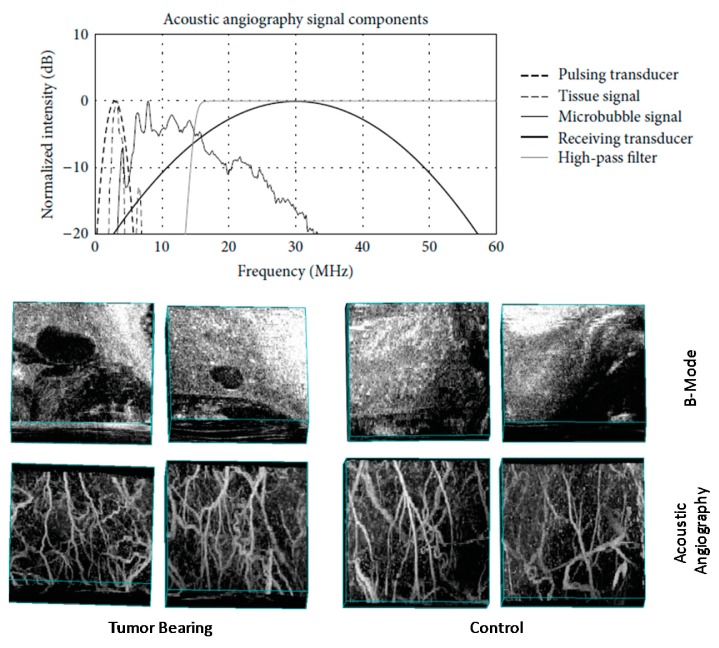
Acoustic angiography amplitude spectrum (**top**) and example images (**bottom**). Wideband separation between tissue response and microbubble response produces images that are drastically different from conventional B-mode images and illustrate blood flow in the microvasculature with high-resolution and high-contrast to tissue ratio. Acoustic angiography images displayed using maximum intensity projections of volumetric scan volumes. Bounding boxes are approximately 0.75 × 1.25 × 1.5 cm (axial, lateral, and elevational). Figure adapted from [68].

Using this technology, Foster's group working with Dayton has constructed several mechanically-steered transducers consisting of concentric low- (2.5–4 MHz) and high-frequency (25–30 MHz) elements [64,65]. These probes have been integrated with a commercial small animal imaging system (VisualSonics, Toronto, ON, Canada) (Figure 2). Imaging with these dual-frequency transducers has provided a high-resolution (∼200 μm), high CTR (∼25 dB) imaging technique, which the authors call “acoustic angiography” due to the resemblance between the vascular images acquired and those in x-ray, or magnetic resonance angiography [66]. This approach has demonstrated sensitivity to vessels containing contrast agents at frequencies higher than previously published (as high as 10 times the transmission frequency) (Figure 3) [67]. As a result, acquired images can be segmented by computational algorithms to analyze vessel morphology based on quantitative metrics such as vessel density and tortuosity [67–69].

**Figure 4.**
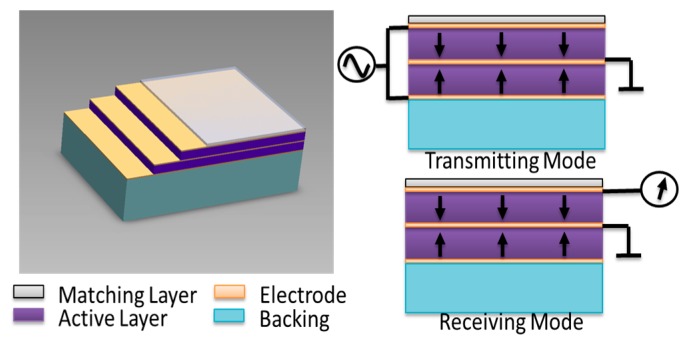
Schematic view of a dual-layer, dual-frequency transducer (**left**) and its operation for transmitting and receiving (**right**). When transmitting, both active layers are electrically connected in parallel and are excited by the same signal, effectively behaving as a single, active element at *f_0_*. When receiving, the front layer records the majority of the signal with a resonance at twice the transmission frequency (2*f_0_*) because the thickness of the active layer has effectively been halved. Figure reprinted with permission from [71].

Several similar transducers have recently been reported. A mechanically-scanned transducer with two concentric elements operating at 4 MHz and 14 MHz was demonstrated by Guiroy *et al.* [70]. Li *et al.* have alternatively demonstrated a micromachined PMN-PT 1–3 composite based dual-frequency (17.5/35 MHz) transducer (Figure 4) for harmonic imaging [71]. In this design, two active layers were mechanically bonded in series and poled in opposite directions. Composite piezoelectrics and electrostrictive materials such as PMN-PT have been increasingly utilized over traditional ceramics like PZT, which have limitations for use at higher frequencies due to manufacturing challenges and grain dimensions which become increasingly close to one wavelength as frequency increases [72].

In recent years, researchers have developed several other dual-frequency transducers with high receiving frequencies (>10 MHz) for specific applications. One area of interest has been intravascular ultrasound, in which a small catheter-based transducer is introduced into the body to perform minimally invasive imaging of occlusive plaques within the coronary arteries [73]. In particular, the presence of vessels known as *vasa vasorum* (70–180 μm in diameter [74]) in plaques has been hypothesized to be linked with decreased plaque stability and increased risk of future complications [75]. Because superharmonic imaging of microbubbles could enable direct visualization of *vasa vasorum*, several researchers have pursued dual-frequency transducer designs for this application. In 2005, Vos *et al.* developed single element transducers for second harmonic imaging designed with dual resonance peaks at 20 and 40 MHz and a 1 mm outer diameter [76,77]. This group has also used commercial IVUS catheters to demonstrate the benefits of pulse inversion detection methods for this application [78,79].

**Figure 5.**
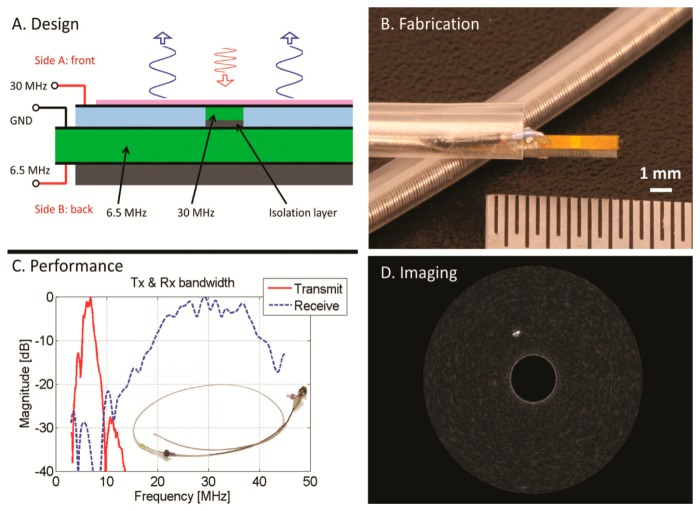
Dual-frequency IVUS transducer designed for superharmonic imaging. (**A**) Structure of the transducer with stacked dual layers of piezoelectric plates; (**B**) The fabricated prototype mounted in an 8.5 F commercial catheter; (**C**) Use of two elements with separate bandwidths allows coverage of the transmitting and receiving frequencies needed for the superharmonic imaging; (**D**) Contrast enhanced IVUS imaging with microbubbles in phantom with fully-developed speckle. Figure reprinted with permission from [80], front cover.

Recently, Jiang's group working with Dayton reported on the design and evaluation of dual-frequency IVUS transducers for superharmonic imaging (6.5 MHz/30 MHz) which were fabricated using stacked piezoelectric plates (Figure 5A) with co-aligned transmit and receive beams [80]. Transmitting and receiving elements were electrically separated by a frequency selective isolation layer (FSIL) [81] between the two active elements. This transducer had an aperture size of 0.6 mm × 3 mm and could be housed inside a commercial (Boston Scientific, Natick, MA) catheter (Figure 5B). Broadband emission and reception were performed by 6.5 and 30 MHz elements, respectively (Figure 5C). Superharmonic IVUS phantom imaging was acquired with a good CTR (12 dB) and high-resolution (200 μm) *in vitro* (Figure 5D).

### 2.2. Other Applications

Researchers have developed dual-frequency ultrasound arrays for applications other than contrast agent imaging, which have provided useful insights into design or fabrication strategies. The earliest reported dual-frequency transducer was that of von Ramm and Smith in 1978, in which a phased array with adjacent rows of 1.5 and 2.5 MHz elements was designed to reduce off-axis contributions to the two-way point spread function by misaligning grating lobes from transmitting and receiving arrays [82]. In 1988, Bui *et al.* showed 1–3 composites can be designed to exhibit multiple frequency sensitivities by tailoring the dimensions for separate resonance modes [83]. De Fraguier *et al.* reported separate transducers for B-mode (4.6 MHz) and color Doppler mode (2.3 MHz) in order to improve Doppler sensitivity by reducing transmitted frequency and increasing pulse length [84]. Similarly, Saitoh *et al.* presented a transducer capable of operating at either 3.75 MHz or 7.5 MHz using two layers of PZT poled in opposite directions for increasing Doppler sensitivity [85].

In 2000, Hossack *et al.* reported a dual-frequency transducer to improve sensitivity for tissue harmonic imaging [86]. This transducer—based on earlier work by Hossack and Auld for increasing bandwidth [87]—used two piezoelectric layers having independent electrical contacts. The two layers were operated either in phase when transmitting at *f_0_*, or 180° out of phase when receiving at 2*f_0_*. Several other authors developed similar transducers having two resonances at the fundamental and twice the fundamental [88,89]. Dual-frequency transducer design in tissue harmonic imaging has been studied extensively in the past and has aided in the development of recent transducer design in contrast specific imaging. For example, confocal annular elements operating at large frequency separations (20 and 40 MHz) produced by Kirk Shung's group were used in tissue harmonic imaging of excised porcine eyes before similar form factor transducers were used in acoustic angiography [90].

In addition, many other authors have demonstrated dual-frequency arrays for combined imaging-therapy applications. Recent devices of interest for imaging have included a three-row array with 128 elements per row for use in prostate cancer imaging and treatment [91], a small-form factor 32-element system for guidance of high-intensity focused ultrasound (HIFU) [92], and a three-row array for thermal strain imaging performing heating at 1.5 MHz and imaging at 5.5 MHz [93]. Smith *et al.* have also demonstrated dual-frequency combined imaging and therapeutic catheter-based devices [94,95]. While a comprehensive review of image guidance in therapeutic ultrasound is beyond the scope of this review, the interested reader is referred to recent reviews on image-guided therapy [96], HIFU [97,98] and thermal strain imaging [99].

Several recent design developments within other applications are of particular interest for imaging microbubble harmonics. Azuma *et al.* have described the design and fabrication of a 0.5/2.0 MHz dual-frequency array for sonothrombolysis and transcranial ultrasound imaging [81]. This paper presented the first dual-frequency array to use a unique design in which the low-frequency array is positioned directly below the high-frequency array within the transducer housing. Low- and high-frequency arrays were isolated by the aforementioned FSIL, an important design achievement. Dual-frequency transducers could also provide distinct advantages over single-frequency transducers in acoustic radiation force impulse (ARFI) imaging, which visualizes mechanical properties of tissue using a low-frequency pushing pulse and high-frequency tracking pulses [100]. Finally, Yen *et al.* have described several dual-layer 2D array transducers capable of performing 3D rectilinear imaging at 5 and 7.5 MHz at a reduced cost relative to conventional 2D arrays with high channel counts [101,102]. Linear and 2D bilaminar arrays with frequency-selective layers could enable imaging of microbubble harmonics over a large field of view with greater image uniformity than that currently afforded by fixed-focus transducers.

## 3. Conclusions

Images formed from harmonic content scattered by ultrasound contrast agents have demonstrated increased image quality over fundamental mode images [103–107], making spectral separation of tissue and harmonic signals using dual-frequency transducers an attractive approach. Transducers having wide frequency separation between the transmission and reception bandwidths have allowed for efficient excitation of microbubbles near resonance and reception of broadband, transient harmonics without dependence on multi-pulse strategies. Single-pulse harmonic imaging has enabled higher frame rates and elimination of motion artifacts found in currently-available contrast imaging approaches requiring multiple pulses. Forming images from higher harmonics has also enabled high-resolution imaging with reduced attenuation, as high-frequency echoes are subject to attenuation in only a single direction. New technical developments have demonstrated the potential for dual-layer, dual-frequency arrays for both 2D and 3D imaging.
